# Adipofascial Flap Monitoring Using Masimo Regional Oximetry: A Case Report and Literature Review on Near-Infrared Spectroscopy for Flap Monitoring

**DOI:** 10.1055/a-2668-3776

**Published:** 2026-01-17

**Authors:** Zhen Luan Low, Nadia Hui Shan Sim, Allen Wei Jiat Wong

**Affiliations:** 1 Plastic, Reconstructive, and Aesthetic Surgery Service, Sengkang General Hospital, Singapore, Singapore

**Keywords:** flap monitoring, regional oximetry, near-infrared spectroscopy, adipofascial flap

## Abstract

Non-invasive monitoring with near-infrared spectroscopy (NIRS) has not been described in the monitoring of non-cutaneous flaps. We present a 29-year-old female with a traumatic left foot injury, where reconstruction was done with a right free adipofascial anterolateral thigh flap. A Masimo O3™ Regional Oximetry pediatric sensor probe was applied directly over the skin-grafted adipofascial flap for continuous flap monitoring and removed at postoperative day 7 uneventfully. We postulate that NIRS does not necessarily require a skin interface, as measurement depends on tissue thickness and penetration of near-infrared wavelengths. A limitation would be the lack of an optimal threshold for regional tissue oxygenation values, but it is reasonable to adopt a threshold of less than 60% absolute value or more than 20% drop from baseline. NIRS has the potential to monitor non-cutaneous flaps as it can detect deep tissue microvascular changes and provide information on tissue perfusion both accurately and non-invasively.

## Introduction

Free flap surgery and flap monitoring have evolved over the years. The prompt recognition of vascular compromise or free flap failure is the most significant factor in impacting successful salvage for a compromised flap. 1 Conventional clinical monitoring of skin color, flap turgor, temperature, capillary refill, and pinprick remains the gold standard of free flap monitoring. However, this is dependent on clinical experience and acumen and varies from institution to institution. 2 Furthermore, clinical monitoring usually requires a skin paddle, which acts as the monitoring flap for examination. 3

Non-invasive monitoring with devices such as near-infrared spectroscopy (NIRS) was first developed for indirect measurement of cerebral perfusion but has since evolved to be used increasingly in free flap monitoring. NIRS utilizes the absorption of infrared light by tissue chromophores contained in hemoglobin to detect real-time changes in tissue perfusion and oxygenation, reported as tissue oxygen saturation (StO _2_ ). 4 Irwin et al. first introduced NIRS as a potential flap monitoring tool by demonstrating experimentally how NIRS was able to detect microcirculatory changes induced by vascular occlusion in rabbit hind limbs. 5

NIRS in postoperative flap monitoring was described in a prospective study of 50 breast reconstructions with free fasciocutaneous flaps, which showed that continuous NIRS monitoring detected vascular compromise before observable clinical signs. 4 A systematic review of studies from 1990 to 2017 similarly concluded that NIRS was effective in detecting subclinical vascular compromise prior to the development of clinical signs in various other flaps. 6 This was further qualified in a recent study of 18 extraoral head and neck reconstructions with free fasciocutaneous flaps, which showed that an absolute decrease of StO _2_ <50% and a sustained decrease of StO _2_ >30% over 1 hour indicated microvascular compromise in 6 out of 18 flaps. 7 Although StO _2_ values differ vastly among different flap types, a systematic review of 15 clinical studies demonstrated high flap success and salvage rates, confirming NIRS as a reliable flap monitoring tool. 8

Despite its versatility in monitoring a variety of flaps, current NIRS technology has been mainly described in fasciocutaneous flaps, but not in non-cutaneous flaps. 4 8 A recent systematic review of 3,529 free flap reconstructions showed that NIRS has only been used in fasciocutaneous flaps. 9 The most commonly used device was the ViOptix T.Ox™ Tissue Oximeter, which uses two wavelengths (690 and 830 nm). 6 9 We describe the use of the Masimo O3™ Regional Oximetry NIRS on an adipofascial anterolateral thigh (ALT) free flap to evaluate the potential for NIRS monitoring of non-cutaneous flaps.

## Case

**Fig. 1:**
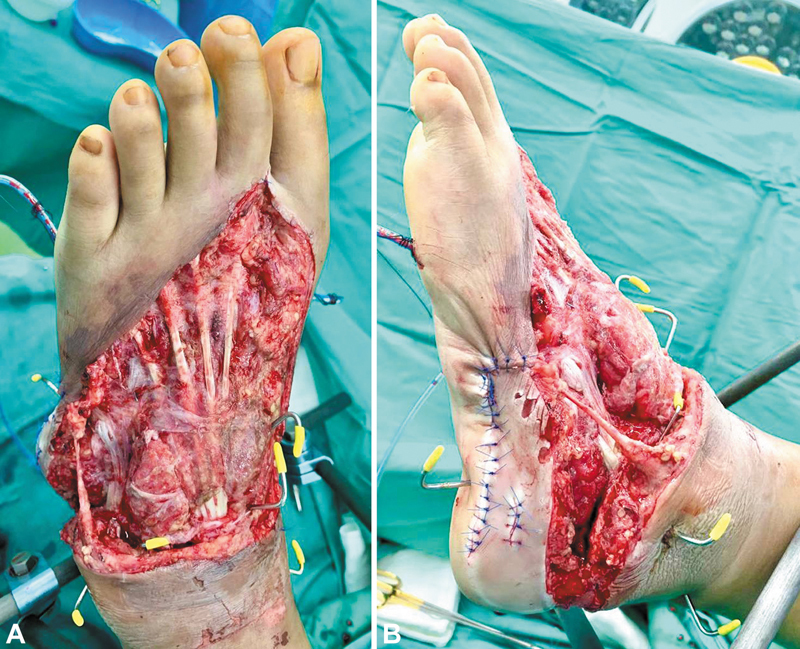
( **A** ) Left foot degloving injury with exposed toe extensor tendons dorsally. ( **B** ) Exposed left lateral ankle joint and calcaneum laterally. Total defect size of 25 cm × 20 cm.

**Fig. 2:**
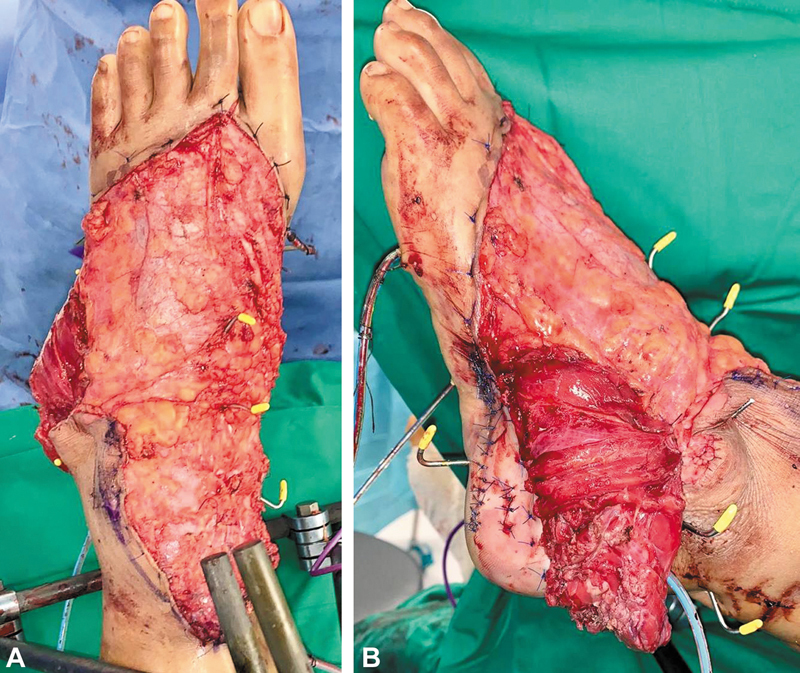
( **A** ) Adipofascial anterolateral thigh flap. ( **B** ) Vastus lateralis component.

**Fig. 3:**
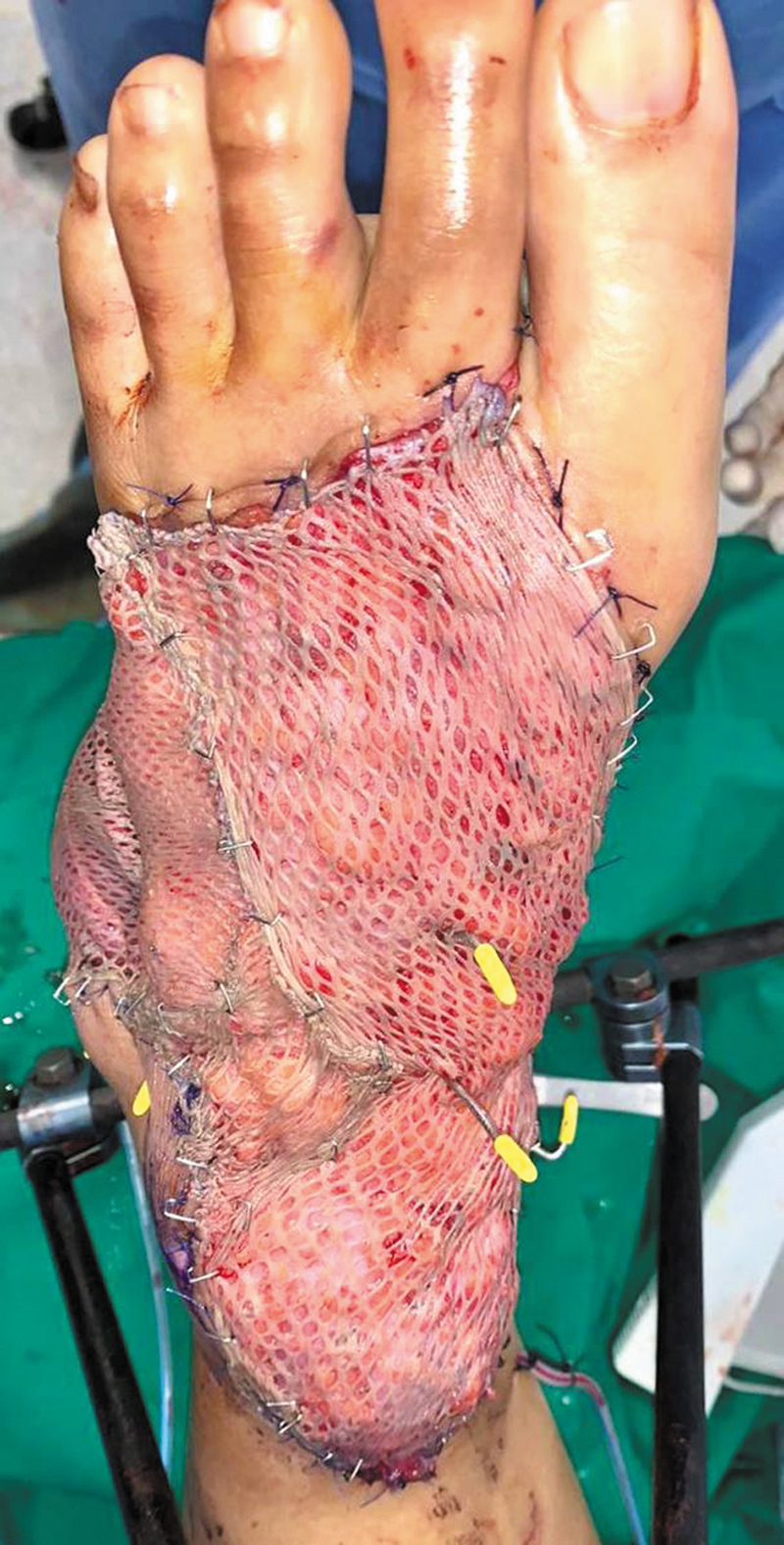
Adipofascial flap with split-thickness skin graft and Kirschner wires.

We present a 29-year-old female who sustained a traumatic open fracture dislocation of her left ankle and calcaneum. There was an extensive degloving soft tissue injury over the left foot dorsum, with a total defect size of 25 cm × 20 cm ( Fig. 1 ). In view of her high body mass index of 33 kg/m ^2^ , a fasciocutaneous ALT flap was unsuitable due to the thick bulk of the flap. Hence, reconstruction was planned with a right free chimeric adipofascial ALT flap with a vastus lateralis component ( Fig. 2 ). The adipofascial flap was thin enough to be perforated to allow flap inset over the Kirschner wires stabilizing the ankle joint and foot dorsum. Split-thickness skin graft (STSG) was harvested from the right medial thigh and meshed to allow passage of the Kirschner wires, then secured over the adipofascial flap ( Fig. 3 ).

**Fig. 4:**
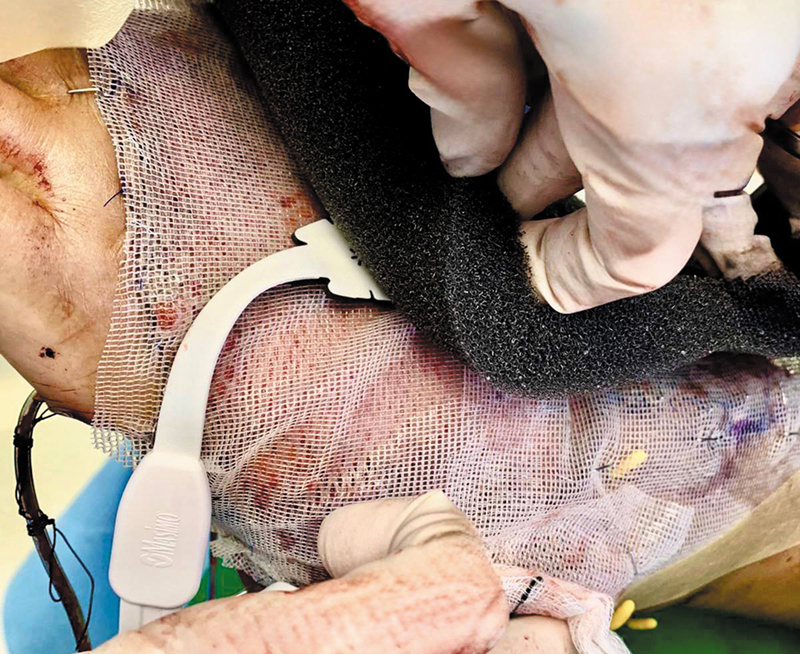
Masimo O3™ Regional Oximetry pediatric sensor probe applied directly over the skin-grafted adipofascial flap for continuous flap monitoring.

**Fig. 5:**
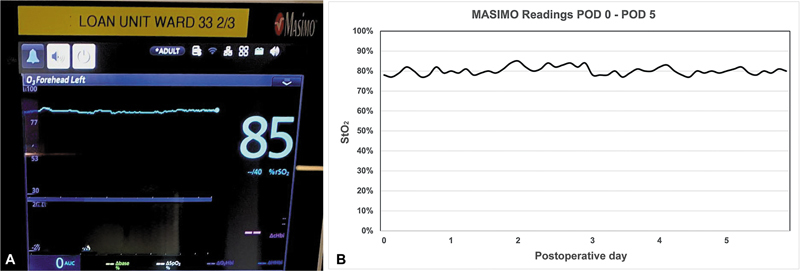
( **A** ) Masimo StO _2_ readings on postoperative day 2. ( **B** ) Graph showing Masimo readings from postoperative day (POD) 0 to POD 5.

**Fig. 6:**
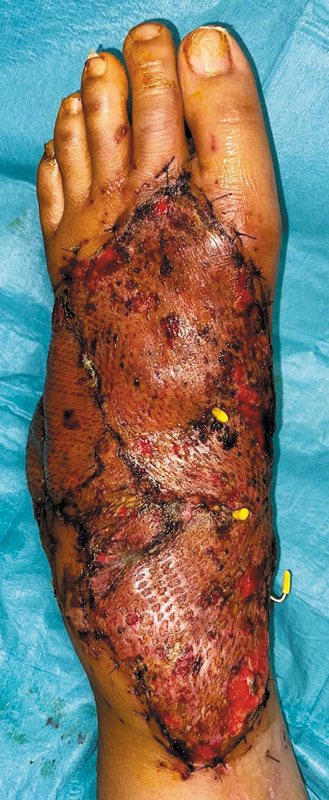
Successful adipofascial flap with good split-thickness skin graft take on postoperative day 14.

A Masimo O3™ Regional Oximetry pediatric sensor probe was applied directly over the STSG for continuous flap monitoring, with measurements taken through the meshed STSG ( Fig. 4 ). The smaller size of the pediatric probe makes it universally suitable for all flap sizes, especially flaps with a smaller monitoring paddle. The O3™ sensor probe consists of an LED emitter and two light detectors for the detection of deep tissue and superficial oxygenation. It uses four wavelengths (730/760/805/880 nm) in the range of near-infrared to measure light absorption in tissue and establish a relationship between light absorbed and tissue oxygen saturation, reflecting tissue regional oxygen saturation. In our Masimo device, the different wavelengths cannot be adjusted and can only be used with the default settings. The sensor was secured onto the flap with a negative pressure wound therapy (NPWT) device set to 125 mm Hg, then connected to an O3™ module, and displayed on the Masimo Root monitor. The initial StO _2_ value was 70 to 80% and ranged from 70 to 85% from immediately postoperatively until postoperative day 7 ( Fig. 5A, B ). We used a threshold of less than 60% absolute value and more than 20% drop from baseline StO _2_ values. The nurses performed hourly monitoring and recording of StO _2_ values. We supplemented NIRS monitoring with conventional flap monitoring methods of clinical observation and pinprick, and the handheld Doppler. There were no instances of flap compromise, and the NIRS monitoring was removed on postoperative day 7. The adipofascial flap remained healthy on postoperative day 14 with the STSG taken with minimal graft loss ( Fig. 6 ).

## Discussion

Early detection of flap compromise is essential in improving flap salvage rates. Clinical observation is the gold standard, but it is unable to monitor flaps without a monitoring skin paddle. Furthermore, it may be limited by flap accessibility and lighting conditions for inspection, and is dependent on the experience of the assessor. 10 Although investigators have tried to circumvent this by creating distal skin paddles, chimeric flaps, or exteriorized flaps as the “monitoring flap” to allow clinical examination, they only provide an indirect indication of the perfusion of the flap of interest. Interpretation can be skewed by vascular compromise of the monitoring flap and must be taken into consideration. 3 There is a need for a reliable adjunct for flap monitoring, and the ideal method of monitoring should be non-invasive, accurate, continuous, and quantitative. The implantable Doppler for buried flaps and ultrasound or laser Doppler for non-buried flaps have been used, but their main limitation would be the lack of continuous monitoring of the flap. 11

NIRS has been well-described in fasciocutaneous flap monitoring, but there is limited knowledge on its utility for flaps without a skin paddle. 5 9 Takasu et al. studied NIRS on a buried latissimus dorsi flap for skull base reconstruction, and suggested that NIRS could be suitable for buried flaps without a skin paddle or skin graft over a muscle flap, given its penetrative depth of 2 to 3 cm. 12 Repez et al. also suggested that NIRS can monitor buried flaps as long as the overlying skin thickness does not exceed the penetration depth of the NIRS sensor. 4

We postulate that NIRS does not necessarily require a skin interface, as measurement depends on tissue thickness and penetration of near-infrared wavelengths. Information is provided on the microcirculatory events occurring in a large volume of tissue and is not limited, as in the case of implantable laser Doppler, to observing pinpoint cutaneous circulatory phenomena. 5 Tanaka et al. showed in their observation of 99 buried deep inferior epigastric perforator flaps that NIRS has a sensitivity of 71.4% and a specificity of 98.9%. NIRS was able to detect abnormal vascularity before the flap monitoring window changed colour. 13 This suggests that NIRS was detecting early vascular changes happening at depths to the cutaneous layer. In our study, the STSG was 8/1,000th of an inch thick, and its contribution to StO _2_ was negligible compared to the volume of the adipofascial flap tissue. This was substantiated with normal StO _2_ values, reflecting tissue oxygen saturation of the flap only.

NIRS allows continuous monitoring and appears to detect microvascular compromise early, making it especially useful in monitoring flaps without a cutaneous component available for clinical monitoring, such as an adipofascial flap. The penetration depth of the NIRS sensor is half of the optode detectors distance. 14 In our study, a probe with 30-mm spacing was used, which corresponded to a penetration depth of 15 mm, suitable for the thin adipofascial flap. Commercially available probes can measure depths ranging from 10 to 30 mm. 8 Furthermore, the non-invasive nature of NIRS means its probe can be repositioned easily and reapplied without the need for recalibration. Therefore, varying the optode detectors distance and hence the penetration depth of NIRS sensors will allow the monitoring of flaps of variable thickness, and especially so for non-cutaneous flaps. The penetrative ability of NIRS suggests that local accumulation of fluid or hematoma beneath the probe would not obstruct accurate monitoring of the flap, assuming that light penetrates to the required depth. 5 In our case, without a skin paddle and the associated thick subcutaneous fat in an obese patient, NIRS was particularly useful in monitoring the thin adipofascial flap.

In our experience with the Masimo NIRS system, it is reliable in detecting both arterial and venous compromise. 9 NIRS is able to provide real-time and objective data of flap perfusion. Chan et al. showed how the Masimo NIRS system provided quantitative evidence of induced ischemia during clamping and return of adequate perfusion to guide the training and division of a pedicled groin flap. 15

NIRS is easy to use, and monitoring can be done with minimal training. This translates to a lesser need for specialized personnel for clinical monitoring, as any member of the team, regardless of clinical experience, can readily identify potential flap compromise and escalate timely. This will be particularly applicable for facilities with a lesser specialized units or manpower for flap monitoring. Hence, NIRS has the potential to be an objective, accurate, and continuous non-invasive flap monitoring technique useful for both cutaneous and non-cutaneous flaps.

NIRS readings can be affected by unstable probe contact and ambient light. 8 Conditions of hypoxemia, such as hypotension and desaturation, could possibly lead to lower NIRS values and a false positive detection. 6 To circumvent the potential limitations, we used an NPWT over the probe to firmly attach it to the flap, remove exudate, and act as a barrier to prevent the penetration of external light. There is a lack of consensus on the optimal threshold for StO _2_ values, as this is flap and device-specific. 8 An observational study of 208 flaps showed a sustained drop of 20% StO _2_ over an hour in 10 compromised flaps, the same criterion threshold for our study. 16 Further studies are needed to establish threshold ranges that can be applied for different NIRS devices and flap types. Nevertheless, continuous NIRS monitoring and observing StO _2_ trends are useful to alert the surgeon early to a compromised flap and help augment clinical judgment.

In conclusion, NIRS has the potential to monitor flaps without a cutaneous component with its ability to detect deep tissue microvascular changes and provide information on tissue perfusion both accurately and non-invasively.
